# A Rare Case of Delayed Cerebral Air Embolism Occurring 7 H After Central Line Removal

**DOI:** 10.1002/ccr3.72200

**Published:** 2026-03-05

**Authors:** Bejan Kanga, Chiedozie Udeh, Parvez Masood, Hayley Andre, David Anthony

**Affiliations:** ^1^ Cleveland Clinic Foundation Cleveland Ohio USA

**Keywords:** air embolism, central venous catheters, critical care, critical care outcomes, foramen ovale, patent, postoperative care

## Abstract

Venous air embolism is a rare complication of central venous catheter insertion that can have devastating consequences. Delayed air entrainment may occur in the presence of fibrin sheath formation via reinforcement of a vein‐to‐dermis fistula, which can lead to air embolism several hours after line removal.

## Graphical abstract

CT demonstrating scattered intracerebral air 7 h after central line removal.

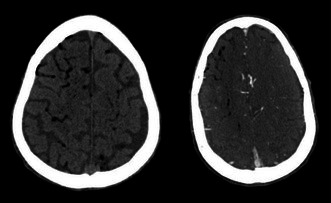

## 1 Introduction

Venous air embolism (VAE) is a rare complication of central venous catheter insertion and removal. The true incidence of VAE is difficult to quantify due to the nonspecific and varied nature of symptoms, with IR literature supporting an incidence of 0.13%, and the reported incidence based on case series ranging from 1 in 47 to 1 in 3000 [1, 2, 3]. In VAE, air entrained into the venous circulation travels to the right heart and subsequently becomes lodged into the pulmonary circulation, leading to hypoxemia, hypercapnia, right ventricular strain, and even cardiopulmonary collapse, depending on the volume of air entrained [1, 2]. Rarely, VAE may lead to arterial air embolism via communication between the venous and arterial systems, as is present in about 25% of the general population with a patent foramen ovale (PFO) [1, 2, 4, 5].

The symptoms of venous and arterial air embolism are usually immediate, with only a few reported cases of delayed presentation. Here we present a case of cerebral air embolism occurring approximately 7 h after central venous catheter removal.

## 2 Case History/Examination

A 64‐year‐old male with a history of severe mitral regurgitation and anxiety was admitted to the cardiovascular ICU following an uncomplicated mitral valve repair and debridement in the setting of infective endocarditis. He had an uncomplicated ICU course, and on postoperative Day 3, in anticipation of transfer to a stepdown unit, underwent bedside removal of a right internal jugular (IJ) single‐lumen 9 Fr introducer and left brachial arterial line. The patient was awake and alert during central line removal, which was performed by the ICU nurse in accordance with institutional protocols (removal during Valsalva maneuver in a supine or Trendelenburg position, followed by immediate pressure to the insertion site with sterile gauze for at least 30 s until hemostasis is achieved, and subsequent application of a petroleum‐based ointment and sterile occlusive dressing).

About 7 h after line removal, the patient began complaining of anxiety and suffered a witnessed panic attack. Shortly afterward, while sitting upright, he had loss of consciousness with associated rightward gaze deviation and hypoxia. There was no loss of spontaneous circulation, but because of persistent obtundation, he was intubated for airway protection.

## 3 Investigations and Treatment

**FIGURE 1:**
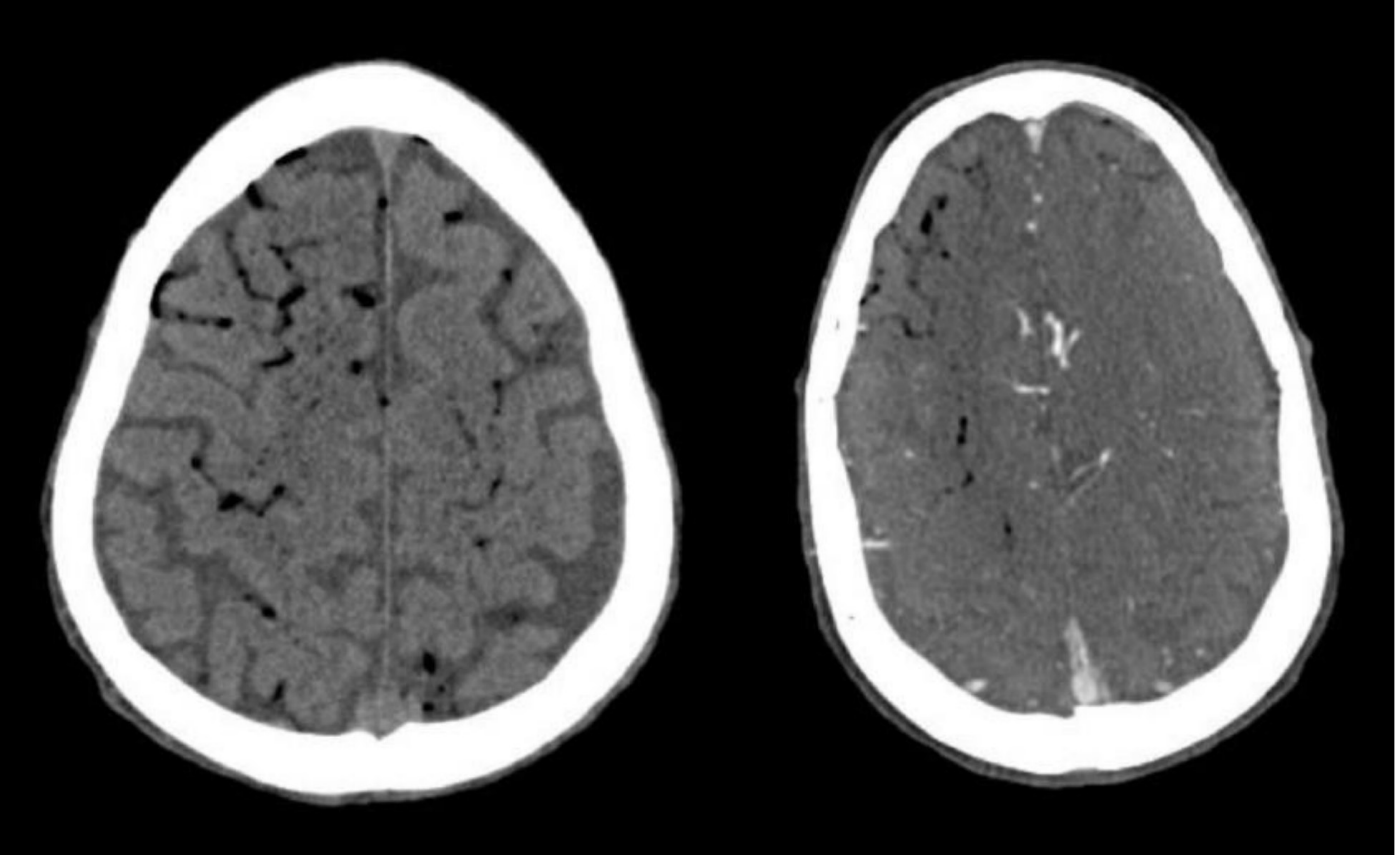
CT demonstrating scattered intracerebral air.

**FIGURE 2:**
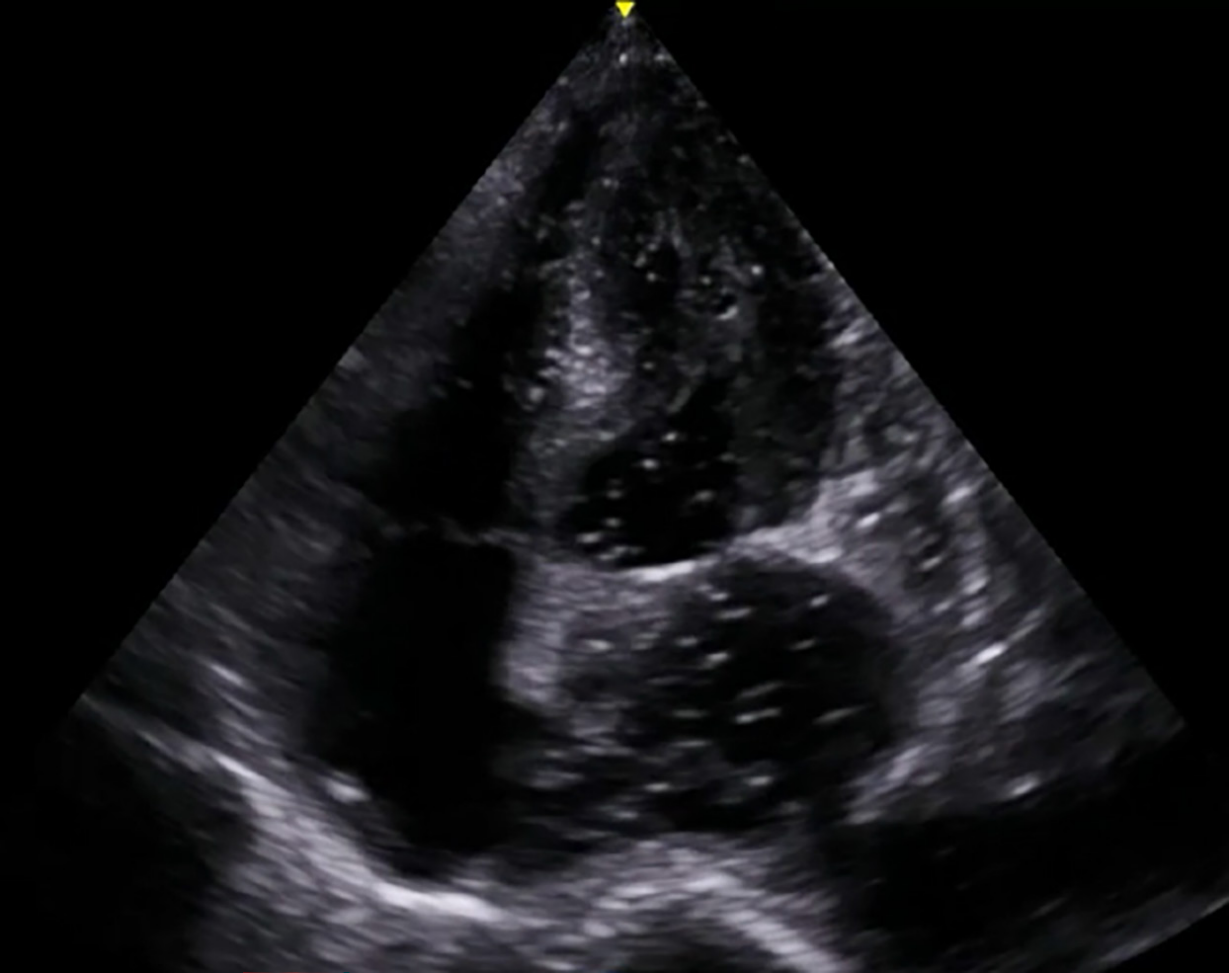
TTE demonstrating air in left atrium and ventricle.

**FIGURE 3:**
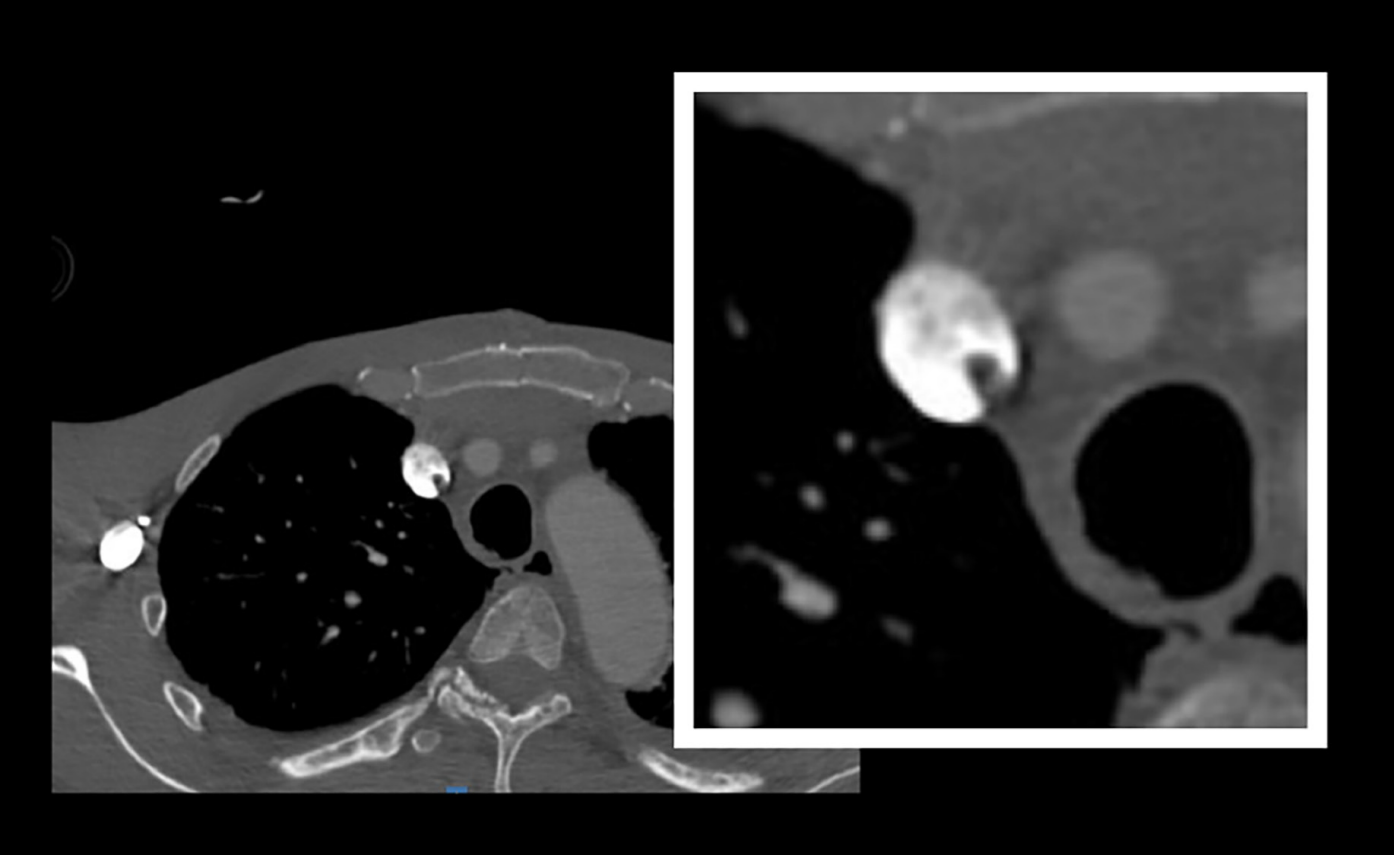
CTA demonstrating fibrin sheath with intraluminal and extraluminal air.

Emergent CT scan of his head demonstrated diffuse air embolism throughout the intracranial vasculature with multiple areas of infarct (Figure 1). Initial bedside transthoracic echocardiogram revealed continued transit of large amounts of air bubbles in both ventricles and IVC (Figure 2), with a small PFO identified on subsequent transesophageal echocardiogram. Ultrasound of the right internal jugular vein revealed echogenicity compatible with thrombus or fibrin sheath, which was confirmed on CTA neck with intraluminal and extraluminal air (Figure 3). Notably, the patient had no arterial access or intravenous infusions at the time of this event, and the occlusive dressing was still in place over the right neck.

The patient was treated with 100% oxygen and placed in the left lateral decubitus and Trendelenburg position for several hours to trap air in the right ventricle. Given the delayed onset of symptoms after line removal and recent surgery, other causes of air entrainment were explored and excluded in the subsequent days, including tracheo‐aortic fistula, perforated intra‐abdominal viscus, and aortic‐esophageal fistula.

## 4 Conclusion

Despite aggressive supportive treatment, the patient had persistent severe neurologic deficits, including depressed level of consciousness, aphasia, and left hemiparesis, with a poor prognosis for recovery. In accordance with his previously expressed preferences, he was transitioned to comfort care measures and died on postoperative Day 24.

## 5 Discussion

Venous air embolism is a known, albeit rare, complication of central line insertion, maintenance, and removal, with air rarely spreading from venous to arterial circulation via a right‐to‐left shunt, such as is present with a PFO [1, 2, 3], leading to simultaneous arterial air embolism. Most cases of central venous catheter‐related VAE are directly related to manipulation or removal of the catheter [1, 2, 3, 4, 6], leading to occlusion of pulmonary, coronary, and/or cerebral vasculature, depending on the nature and quantity of air entrained [1, 2, 7]. Reports of delayed air embolism are very rare, but have been reported, most commonly in the presence of persistent subcutaneous tracts [7, 8, 9, 10].

Fibrin sheaths are commonly associated with central lines and can begin to form immediately after line placement [7, 8, 11]. Rarely, fibrin sheaths can serve as an entry point for air entrainment after line removal via reinforcement of the catheter tract between the skin and vein [7, 8, 9, 10]. This phenomenon may be worsened by thrombus formation [8], as was suggested on ultrasound of our patient, and can lead to delayed VAE.

The risk of air entry after central line removal is increased by several factors, including upright positioning, deep inspiration, talking, and coughing [7, 8, 11]. Therefore, it is recommended that patients be placed in a head‐down position for line removal and instructed to avoid these maneuvers, which increase risk.

If a subcutaneous tract persists, however, it is possible for these maneuvers to elicit air entry even several hours after line removal [7]. We conjecture that upright posture, patient movement, and vigorous hyperventilation during an anxiety attack facilitated air entrainment through the central venous catheter site of our patient, reinforced by the fibrin sheath seen on imaging, leading to VAE and subsequent decline, despite the presence of an occlusive dressing during this period of increased movement.

A high degree of suspicion for venous air embolism is needed when patients display symptoms of cardiopulmonary or cerebral dysfunction following central venous catheter removal. Close attention should be placed on entry site closure and occlusive dressing selection and placement to prevent displacement during routine patient activity. Caregivers should consider maintaining these dressings for up to 72 h after line removal to further minimize the risk of delayed venous air embolism.

## Author Contributions

**Bejan Kanga:** conceptualization, data curation, investigation, writing – original draft, writing – review and editing. **Chiedozie Udeh:** conceptualization, supervision, writing – review and editing. **Parvez Masood:** data curation, visualization, writing – review and editing. **Hayley Andre:** writing – review and editing. **David Anthony:** writing – review and editing.

## Funding

The authors have nothing to report.

## Consent

Written informed consent was provided by the patient's family for publication of information in a medical journal, including consent for clinical images and details of medical care. This consent form can be provided upon reasonable request.

## Conflicts of Interest

The authors declare no conflicts of interest.

## Data Availability

The data that support the findings of this study are available in the supporting information of this article.
